# Therapeutic Strategies against *Leishmania* and *Trypanosoma*

**DOI:** 10.3390/pathogens12101263

**Published:** 2023-10-19

**Authors:** André L. S. Santos, Igor A. Rodrigues, Claudia M. d’Avila-Levy, Cátia L. Sodré, Koert Ritmeijer, Marta H. Branquinha

**Affiliations:** 1 Laboratório de Estudos Avançados de Microrganismos Emergentes e Resistentes (LEAMER), Departamento de Microbiologia Geral, Instituto de Microbiologia Paulo de Góes (IMPG), Universidade Federal do Rio de Janeiro (UFRJ), Rio de Janeiro 21941-902, RJ, Brazil; mbranquinha@micro.ufrj.br; 2 Departamento de Produtos Naturais e Alimentos, Faculdade de Farmácia, Universidade Federal do Rio de Janeiro (UFRJ), Rio de Janeiro 21941-902, RJ, Brazil; igor@pharma.ufrj.br; 3 Laboratório de Estudos Integrados em Protozoologia, Fundação Oswaldo Cruz-Fiocruz, Instituto Oswaldo Cruz, Coleção de Protozoários da Fiocruz, Rio de Janeiro 21040-900, RJ, Brazil; davila.levy@ioc.fiocruz.br; 4 Departamento de Biologia Celular e Molecular (GCM), Instituto de Biologia (IB), Universidade Federal Fluminense (UFF), Niterói 24210-201, RJ, Brazil; catiasodre@id.uff.br; 5 Public Health Department, Médecins Sans Frontières, 1018 DD Amsterdam, The Netherlands; koert.ritmeijer@amsterdam.msf.org

Human African trypanosomiasis (also known as sleeping sickness, with *Trypanosoma brucei gambiense* and *Trypanosoma brucei rhodesiense* as etiological agents), American trypanosomiasis (also known as Chagas disease, with *Trypanosoma cruzi* as the etiological agent), and leishmaniasis (including cutaneous, mucocutaneous, and visceral forms, with multiple species belonging to the *Leishmania* genus as etiological agents) are recognized as neglected tropical diseases (NTDs). These diseases affect marginalized populations and pose a high-impact health problem, primarily in low- or low-to-middle-income countries in Africa, Asia, Latin America, and the Caribbean [1,2,3]. *Leishmania* and *Trypanosoma* not only infect humans, but they also infect wild and domesticated animals, which serve as reservoirs for these diseases [1,4]. Relevantly, the movement of people and animals across borders and within countries has become increasingly common in our interconnected world, and this mobility can both facilitate the transmission of diseases and challenge efforts to control outbreaks [1,5]. Furthermore, climate changes can contribute to the spread of NTDs to areas that were previously unaffected [6].

Chagas disease, sleeping sickness, and leishmaniasis register the highest rates of mortality amongst all NTDs [1,4]. In this context, chemotherapy remains the first strategy, which aims to control and eliminate these illnesses. However, the current therapeutic interventions to treat these NTDs are inadequate, using poorly effective drugs that are often associated with several severe side effects to the patient, as well as inconvenient routes of administration, which, in some cases, result in the abandonment of the treatment protocol [7,8,9]. In addition, the emergence of drug-resistant parasites is increasing worldwide [7,8,9]. To worsen this scenario, the development of new promising therapies to combat NTDs is severely limited by insufficient funding and the lack of governmental incentive programs. Moreover, pharmaceutical companies are not interested in developing novel effective drugs for NTDs in a timely manner due to the lack of significant profit inducements [7,8,9]. So, it is crucial for the international scientific community to develop novel drugs and new strategies to combat NTDs, which are essential actions for improving the lives of socioeconomically deprived individuals who are affected by these diseases [10,11]. 

The present “Topic”, published jointly by both the *Tropical Medicine and Infectious Disease* and *Pathogens* journals, titled “Novel Therapeutic Strategies against *Leishmania* and *Trypanosoma*”, can be considered a feasible platform to disseminate new information, perspectives, and viewpoints on these NTDs.

The “Topic” is composed of four research articles on both *Leishmania* and *Trypanosoma* infectious agents. In order to provoke the reader’s interest, a brief overview of each published paper is provided as follows: 

The first paper, written by Ubals and colleagues [12], described a retrospective study (from 2012 to 2018) conducted at Vall d’Hebron University Hospital (Barcelona, Spain) on the treatment of complex cases of cutaneous leishmaniasis (caused by *Leishmania infantum* or *Leishmania major*) with the intravenous administration of liposomal formulations of amphotericin B. Interestingly, the authors reported the cure of all 16 patients within the first three months post-treatment, and no relapses were reported at the 12-month follow-up.

The second paper, published by Branquinha and colleagues [13], reported the in vitro efficacy of the calpain inhibitor MDL28170 when combined with amphotericin B against both the promastigote and amastigote forms of both *Leishmania amazonensis* (the etiological agent of cutaneous leishmaniasis) and *Leishmania infantum* (the etiological agent of visceral leishmaniasis). The authors also proposed possible mechanisms of action by using multiple morphological and biochemical approaches.

In the third paper, Suganuma and colleagues [14] demonstrated the in vivo therapeutic potency and efficacy of orally administered nitrofurantoin—an antimicrobial agent that is commonly used to treat human bacterial urinary tract infections—in BALB/c mice who had been intraperitoneally infected with *Trypanosoma congolense* (the etiological agent of animal African trypanosomiasis or Nagana).

The fourth paper, published by Garcia and colleagues [15], reported the anti-*Leishmania amazonensis* and anti-*Leishmania infantum* activity of the naturally occurring alkaloid tryptanthrin. The authors described tryptanthrin’s potent action on both the promastigote and amastigote forms, as well as its ability to trigger apoptosis-like cell death in both *Leishmania* species. Additionally, the in silico pharmacokinetics and toxicological properties of tryptanthrin were explored.

The editors hope that this “Topic” can be useful for students, teachers, researchers, and clinicians. Indeed, the editors firmly believe that developing tools and strategies to stimulate the search for new and promising antimicrobials is an imperative task for the international scientific community that should be continuously pursued and advanced, as such initiatives will not occur spontaneously. To finalize, the editors are extremely grateful to all contributing authors for their enthusiasm and valuable cooperation, and to the consulting editors for their expert and exhaustive scientific reviews.
